# Risk of dementia or cognitive impairment in COPD patients: A meta-analysis of cohort studies

**DOI:** 10.3389/fnagi.2022.962562

**Published:** 2022-09-09

**Authors:** Jun Wang, Xuanlin Li, Siyuan Lei, Dong Zhang, Shujuan Zhang, Hailong Zhang, Jiansheng Li

**Affiliations:** ^1^Co-Construction Collaborative Innovation Center for Chinese Medicine and Respiratory Diseases by Henan and Education Ministry of P.R. China, Henan University of Chinese Medicine, Zhengzhou, China; ^2^Henan Key Laboratory of Chinese Medicine for Respiratory Disease, Henan University of Chinese Medicine, Zhengzhou, China; ^3^Department of Respiratory Diseases, The First Affiliated Hospital of Henan University of Chinese Medicine, Zhengzhou, China

**Keywords:** chronic obstructive pulmonary disease, dementia, cognitive impairment, meta-analysis, systematic reviews

## Abstract

**Purpose:**

A meta-analysis of cohort studies was performed to evaluate the association between COPD and the risk of dementia or cognitive impairment.

**Methods:**

Cohort studies that evaluated the association between COPD and the risk of dementia or cognitive impairment were identified by a systematic search of PubMed, Embase, Web of Science, and Cochrane Library databases. The search time frame was from database establishment to April 12, 2022, with two reviewers independently screening the literature and extracting data. The Newcastle-Ottawa Quality Assessment Scale (NOS) was used to conduct the quality evaluation. Then, a meta-analysis was performed using Stata 15.1 software.

**Results:**

Six cohort studies including 428,030 participants were included. The overall quality of the included studies was high, with an average NOS score of over 7. Meta-analysis showed that compared to those without COPD at baseline, patients with COPD were associated with a significant increased risk of dementia (*RR* = 1.24, 95% *CI* = 1.03 ~ 1.50, *I*^2^ = 96.6%, *z* = 2.25, *p* = 0.024) and cognitive impairment (*RR* = 1.30, 95% *CI* = 1.13 ~ 1.49, *I*^2^ = 50.1%, *z* = 3.72, *p* < 0.001). Subgroup analysis suggested no significant difference in the risk of dementia among COPD patients of different genders. Nevertheless, in terms of age, the risk of dementia varied among COPD patients of different ages, which was most distinguished in patients younger than 65 years.

**Conclusion:**

COPD patients have a higher risk of developing dementia or cognitive impairment compared to those without COPD, and this risk is not affected by gender but seems to be associated with age.

**Systematic review registration:**

https://www.crd.york.ac.uk/prospero/, identifier CRD42022325832.

## Introduction

Chronic obstructive pulmonary disease (COPD) is a leading cause of death and disability worldwide (World Health Organization, 2019; Christenson et al., 2022). According to reports, the number of COPD patients worldwide reached 212.3 million in 2019, resulting in 3.3 million deaths, and the prevalence will continue to rise, by 2060, more than 5.4 million may die from COPD and related diseases (Global Initiative for Chronic Obstructive Lung Disease, 2021; Adeloye et al., 2022; Safiri et al., 2022). COPD often coexists with other diseases (comorbidities) that may have a significant impact on disease course and contribute significantly to a reduction in quality of life and the economic cost of the disease (Tsantikos et al., 2018).

Cognitive impairment is one of the common comorbidities of COPD. The Global Initiative for Chronic Obstructive Lung Disease (GOLD) stated that the average prevalence of cognitive impairment in COPD is 32% (Global Initiative for Chronic Obstructive Lung Disease, 2020). Research revealed COPD patients with comorbid cognitive impairment are more likely to suffer from adverse factors such as difficulties with daily functioning, increased risk of dementia, and increased risk of hospitalization and mortality (Chang et al., 2012; Dodd et al., 2013; Dodd, 2015; Baird et al., 2017). Dementia is regarded as a global public health problem, and the number of people with dementia is projected to increase from around 57 million globally in 2015 to 152 million by 2050 (GBD 2019 Dementia Forecasting Collaborators, 2022). Projected global costs could grow to $2 trillion by 2030, with detrimental implications for social and healthcare systems (Wimo et al., 2017). COPD patients with dementia were associated with higher rates of systemic comorbidities, longer hospital stays, and higher hospital costs (Jackson et al., 2020; Gupta et al., 2022).

Currently, there is already some evidence indicating COPD is an important factor in the increased risk of dementia or cognitive impairment (Li et al., 2013; Samareh Fekri et al., 2017; Lutsey et al., 2019). Nevertheless, some latest research revealed inconsistent conclusions (Cherbuin et al., 2019; Siraj et al., 2020). Some common risk factors exist between COPD and dementia or cognitive impairment, such as aging (Brayne et al., 2006; Blazer and Wu, 2009; Savale et al., 2009), inflammation (Li et al., 2020), and smoking (Okusaga et al., 2013), which causes difficulty in judging whether there are co-existence of conditions or true association. There is still a lack of consensus regarding the association between COPD and dementia or cognitive impairment, and the exact nature between COPD and the risk of dementia or cognitive impairment remains uncertain. To determine this relationship, we conducted this meta-analysis of the cohort studies to present whether COPD patients are associated with an increased risk of dementia or cognitive impairment.

## Methods

This study was reported in accordance with the Meta-analysis of Observational Studies in Epidemiology (MOOSE) (Stroup et al., 2000) and the Preferred Reporting Items for Systematic Reviews and Meta-Analyses (PRISMA) guidelines (Page et al., 2021). The protocol has been pre-registered in the International Prospective Register of Systematic Reviews (PROSPERO) (CRD42022325832).

### Data source and searches

We systematically searched the PubMed, EMBASE, Cochrane Library, and Web of Science databases without language restrictions from their inception to April 12, 2022. The Medical Subject Headings (MeSH) terms and keywords used in the search were as follows: (“Pulmonary Disease, Chronic Obstructive” OR “Pulmonary Disease, Chronic Obstructive” OR “chronic obstructive lung disease” OR “COPD” OR “COAD” OR “chronic obstructive pulmonary disease” OR “chronic obstructive airway disease” OR “chronic obstructive respiratory disease” OR “Chronic bronchitis” OR “chronic emphysema” OR “chronic airflow obstruction”) AND (“dementia” OR “Alzheimer's disease” OR “vascular dementia” OR “multiinfarct dementia” OR “cognition disorders” OR “cognitive defect” OR “cognitive decline” OR “cognitive deficit” OR “cognitive dysfunction” OR “cognitive impairment” OR “mild cognitive impairment” OR “neurocognitive disorder” OR “memory impairment”). The references of the included studies and existing systematic reviews were hand-searched to find additional relevant articles. The full search strategy was included in Supplementary Appendix 1.

### Eligibility criteria

The included studies were required to meet the following criteria: (1) cohort study design; (2) the exposed group consisting of patients with confirmed COPD diagnosis, and the control group consisting of patients without COPD; (3) the risk of dementia or cognitive impairment as the outcome, expressed as an adjusted Odds Ratio (*OR*), Relative Risk (*RR*) or Hazard Ratio (*HR*).

### Exclusion criteria

Exclusion criteria were as follows: (1) conference abstracts or study protocols; (2) duplicate publications; (3) studies with incomplete data or no relevant outcome.

### Research selection

Five reviewers (XL, SL, DZ, JW, and SZ) independently screened the literature. Duplicate and irrelevant articles were first excluded according to their titles and abstracts. Thereafter, the full texts of the potentially eligible articles were downloaded and read to identify all eligible studies. In case of disagreement, discussions were conducted with an independent adjudicator (JL) until a consensus was reached.

### Data extraction

Four reviewers (JW, XL, HZ, and JL) independently extracted the following data using predesigned forms according to the guideline for data extraction for systematic reviews and meta-analysis (Taylor et al., 2021), including the following information: first author, year of publication, country, study type, sample size, study period, follow-up years, age of participants, diagnosis of COPD and dementia or cognitive impairment, stage of COPD, dementia type, adjusted confounder, et al. For a study with multiple *RR/OR/HRs* provided by different analytical models, we selected suitable *RR/OR/HRs* based on whether their confounders adjusted were similar to other studies. In the results section, all relative effects metrics were referred to as *RRs*, which did not affect our results or their interpretation (Dahabreh et al., 2012).

### Risk-of-bias assessment

The Newcastle-Ottawa Quality Assessment Scale (NOS) (Stang, 2010) was used to assess the quality of the included studies in three aspects, namely, selection, comparison, and results. The scores of cohort studies ranged from 0 to 9. Higher scores indicated a higher research quality; specifically, NOS scores of ≥7, 4 ~ 6, and 0 ~ 3 indicated high, moderate, and low quality, respectively.

### Statistical analysis

The adjusted *RR/OR/HRs* and 95% *CI* from each study were used to assess the risk of dementia or cognitive impairment in COPD. We assessed heterogeneity using the chi-square test and *I*^2^ value, and *p* < 0.1 or *I*^2^> 50% was considered to indicate heterogeneity; in such instances, the random-effects model was adopted. Otherwise, the fixed-effects model was employed. We performed sensitivity analyses to verify the robustness of the overall results and explore the sources of heterogeneity. Since few studies were included, we only performed subgroup analysis on gender and age. Considering the differences in physiological structure and living habits of COPD patients of different genders may be an important factor affecting the risk of dementia. Furthermore, age as a risk factor for dementia and COPD may have a significant impact on the relationship between them. All statistical analyses were performed using the Stata software (version 15.1).

## Results

### Literature search

**Figure 1:**
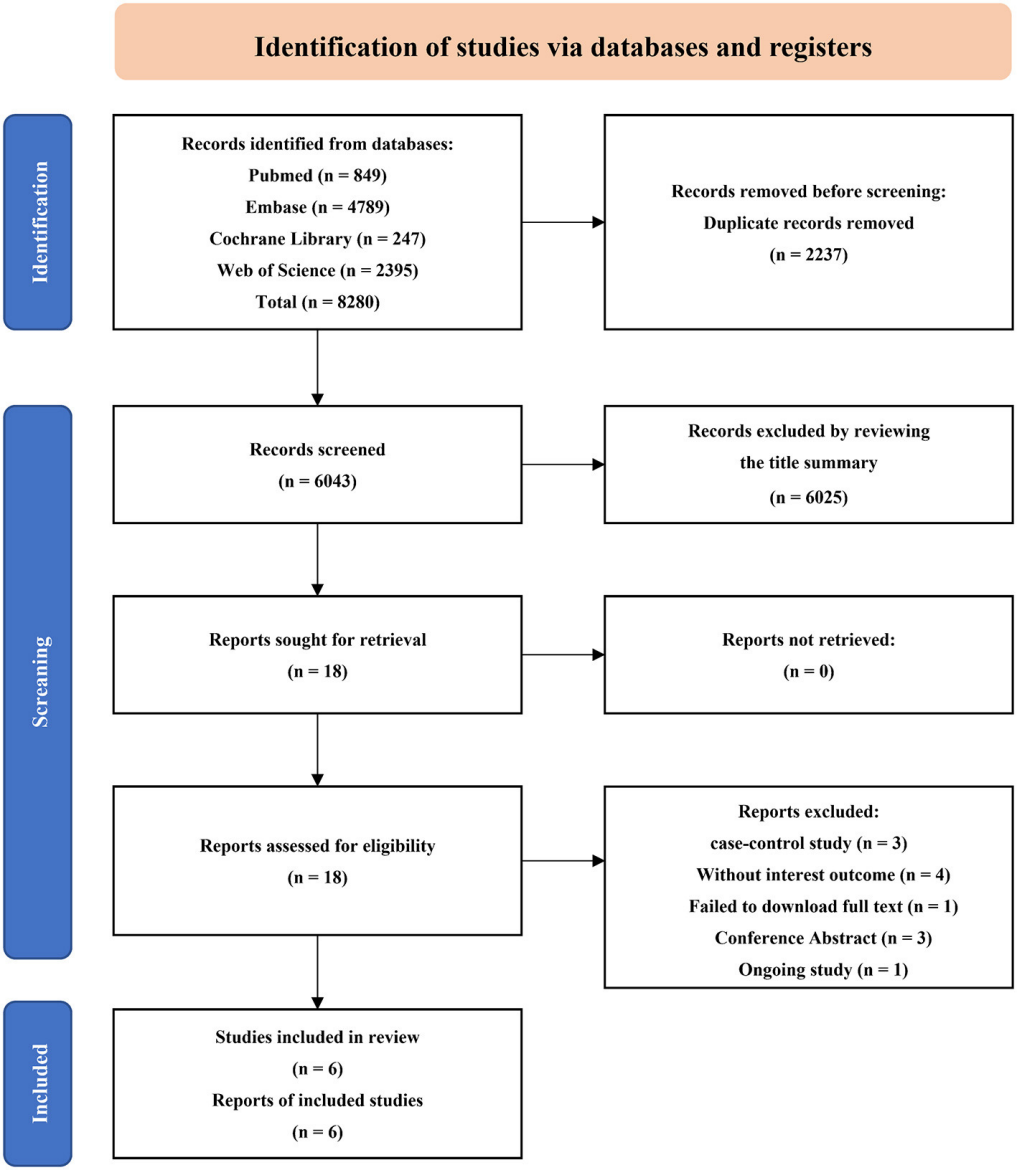
Flowchart of literature search and study selection.

A total of 8,280 articles were identified from the initial search, of which 2,237 duplicate articles were excluded (machine check: *n* = 1,582; manual check: *n* = 655). Furthermore, 6,025 articles were excluded from a two-step screening according to the title and abstract, firstly, the literature that focused on diseases was obviously inconsistent with this study was eliminated (*n* = 4,845); secondly, those that did not match the research topic and type were eliminated through careful review (*n* = 1,180). Then 18 articles after full-text reading, including case-control study (*n* = 3), conference abstracts (*n* = 3), study with no interested outcome (*n* = 4), full text failed to download (*n* = 1) (Feng et al., 2016), and ongoing study (*n* = 1) (Thakur et al., 2010). Finally, 6 studies were included in this review. The search selection process is shown in Figure 1, and the information on excluded studies is included in Supplementary Appendix 2.

### Characteristics of studies

This meta-analysis included 6 cohort studies covering 428,030 individuals. Of these, five studies (Liao et al., 2015a,b; Xie and Xie, 2019; Siraj et al., 2020; Tao et al., 2020) were retrospective cohort studies, while the other one (Singh et al., 2014) was prospective cohort studies. These studies were published between 2014 and 2021. The sample size for each study ranged from 1,425 to 307,817 individuals. The mean age of the participants in the included studies ranged from 65.70 to 82.91, and the average follow-up time ranged from 2.8 to 10.2 years. Four studies (Liao et al., 2015a,b; Xie and Xie, 2019; Tao et al., 2020) were conducted in China, one study (Singh et al., 2014) was conducted in the United States of America (USA), and one (Siraj et al., 2020) in the United Kingdom (UK). The adjusted estimates were available for almost all studies even though the adjusted confounders are slightly different. The main characteristics of the included studies are shown in Table 1.

**Table 1:** Characteristics of studies included in the review.

**Author (year)**	**Country**	**Study type**	**Sample size total (case/control)**	**Follow up years**	**Age (years) case/control**	**Diagnosis of COPD**	**Diagnosis of dementia/cognition impairment**	**Dementia/cognition impairment Type**	**Confounders adjusted**
Siraj et al. (2020)	UK	Retrospective cohort study	307,817 (64,397/243,420)	4.4 average	66.40 ± 10.90 65.70 ± 11.00	Read-coded COPD diagnosis	Read codes in consultation with a geriatrician	Dementia/CI/Vascular Dementia	Age, gender, Townsend social deprivation score, smoking status, MRC, CCI
Tao et al. (2020)	China	Retrospective cohort study	26,876 (13,438/13,438)	7	74.88 ± 6.72	ICD-9-CM codes: 490–492, 494, 496	ICD-9-CM codes: 290.40–290.43	Alzheimer's disease	Sex, age, insurance status, payroll bracket
Xie and Xie (2019)	China	Retrospective cohort study	4,735 (515/4,220)	3	82.91 ± 9.74	Self-report	MMSE score: <17(illiterate participants); <20(1–6 years of education); <24(over 6 years of education)	MCI/Dementia	Age, gender, marital status, education level, alcohol drinking, current exercise, baseline BMI, baseline prevalence of hypertension, diabetes, stroke
Liao et al. (2015a)	China	Retrospective cohort study	25,920 (8,640/17,280)	NA	68.76 ± 10.74	ICD-9-CM codes: 490–492, 496	ICD-9-CM codes: 331, 332	Alzheimer's disease	Age, gender, coronary artery disease, stroke, hyperlipidemia, hypertension, diabetes, head injury
Liao et al. (2015b)	China	Retrospective cohort study	61,257 (20,492/40,765)	2.8 ~ 10.2	67.00 ± 12.50 68.20 ± 12.40	ICD-9-CM	ICD-9-CM codes: 290, 294.1, 331.0	Dementia	Sex, age, number of comorbidities
Singh et al. (2014)	USA	Prospective cohort study	1,425 (171/1,254)	3.8 ~ 5.4	79 80	ICD-9, HICDA codes: 491, 492, 496	Physician evaluation (medical history review, neuropsychological testing, administration of the short test of mental status, the unified Parkinson's disease rating scale)	Any MCI/Amnestic MCI/Non- Amnestic MCI	Education, sex, age

### Methodology quality assessment

The NOS scale was used to assess the quality of the included studies, and the results are shown in Table 2. Three studies (Liao et al., 2015a; Siraj et al., 2020; Tao et al., 2020) had a score of 8 and three studies (Singh et al., 2014; Liao et al., 2015b; Xie and Xie, 2019) had a score of 7, which were classified as high quality. The mean score of the six studies was 7.5, indicating an overall high quality.

**Table 2:** Quality of cohort studies in this review.

**Author, year**	**Selection**	**Comparability**	**Outcome**	**Total score**
Siraj et al. (2020)	⋆⋆⋆⋆	⋆⋆	⋆⋆	8
Tao et al. (2020)	⋆⋆⋆⋆	⋆⋆	⋆⋆	8
Xie and Xie (2019)	⋆⋆⋆	⋆⋆	⋆⋆	7
Liao et al. (2015a)	⋆⋆⋆⋆	⋆⋆	⋆⋆	8
Liao et al. (2015b)	⋆⋆⋆⋆	⋆	⋆⋆	7
Singh et al. (2014)	⋆⋆⋆⋆	⋆	⋆⋆	7

### Association between COPD and the risk of dementia

**Figure 2:**
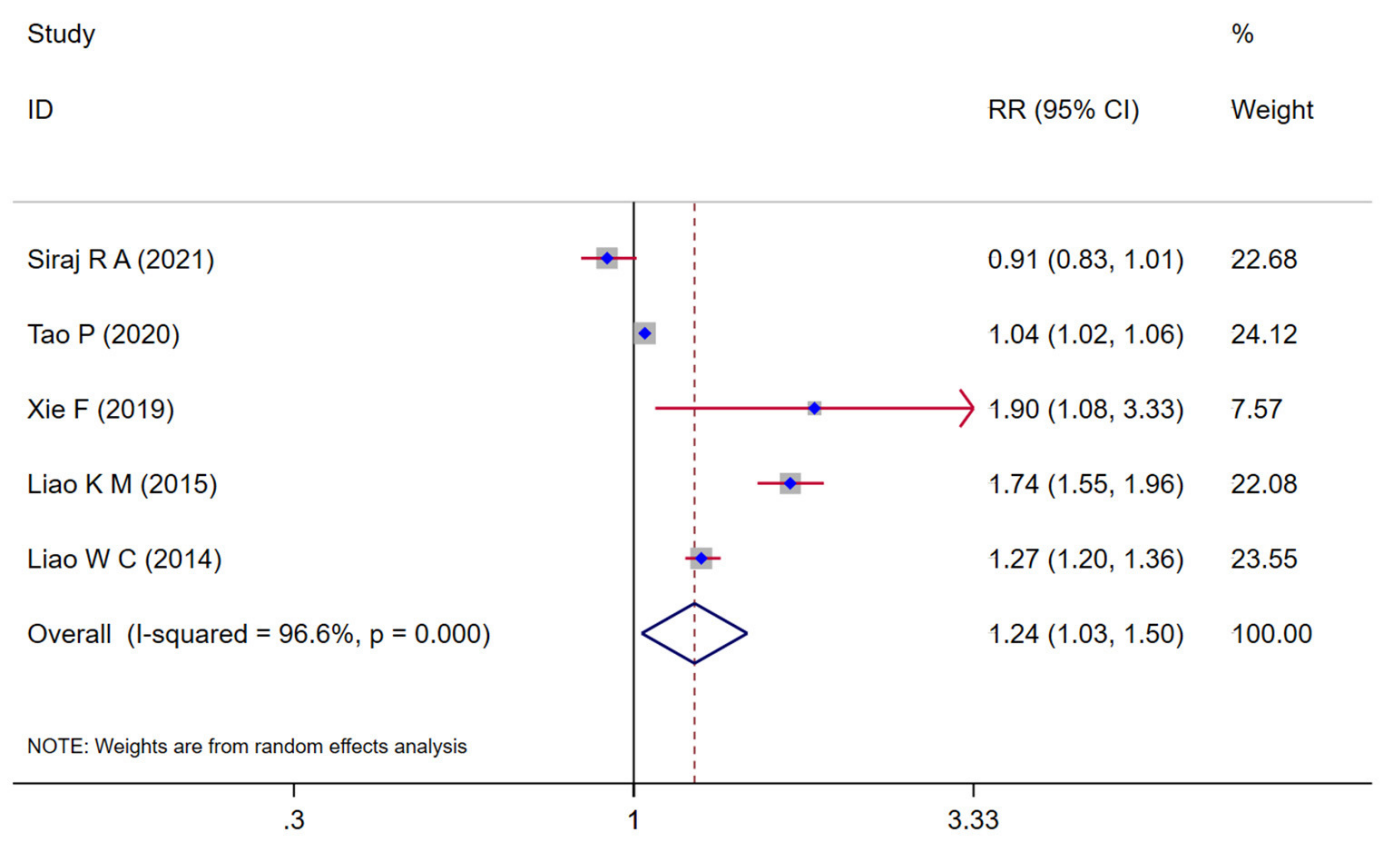
Forest plot showing the effect of COPD on dementia.

A total of five studies (Liao et al., 2015a,b; Xie and Xie, 2019; Siraj et al., 2020; Tao et al., 2020) assessed dementia as an outcome, of them, four studies (Liao et al., 2015a,b; Xie and Xie, 2019; Tao et al., 2020) showed that COPD patients is associated with a subsequent higher risk of dementia ranging from (*RR* = 1.04, 95% *CI* = 1.02 ~ 1.06, *p* = 0.002) to (*RR* = 1.90, 95% *CI* = 1.08 ~ 3.33, *p* = 0.026); one latest study (Siraj et al., 2020) showed that incidence of dementia was not as frequently recorded in patients with COPD (*RR* = 0.91, 95% *CI* = 0.83 ~ 1.01, *p* = 0.053). The pooling analysis shows that COPD was associated with an increased risk of dementia (*RR* = 1.24, 95% *CI* = 1.03 ~ 1.50, *I*^2^ = 96.6%, *z* = 2.25, *p* = 0.024; Figure 2). Notably, since the sample size was significantly smaller than other studies and the confidence intervals were wide, the weight of the study performed by Xie et al is less than the other study. Owing to the significant heterogeneity (*I*^2^ = 96.6%, *p* < 0.001), we performed a sensitivity analysis by omitting each study to explore the source of heterogeneity and found that the results were identical to the primary results after removing any one of the studies one at a time. This indicated that the overall results were relatively robust, no single study exerted a substantial influence on the pooled *RR* Supplementary Appendix 3. Unfortunately, although we excluded studies one by one from the analysis, it was not effective in reducing heterogeneity. The funnel plot was not implemented due to fewer studies (<10).

### Gender of COPD and risk of dementia

**Figure 3:**
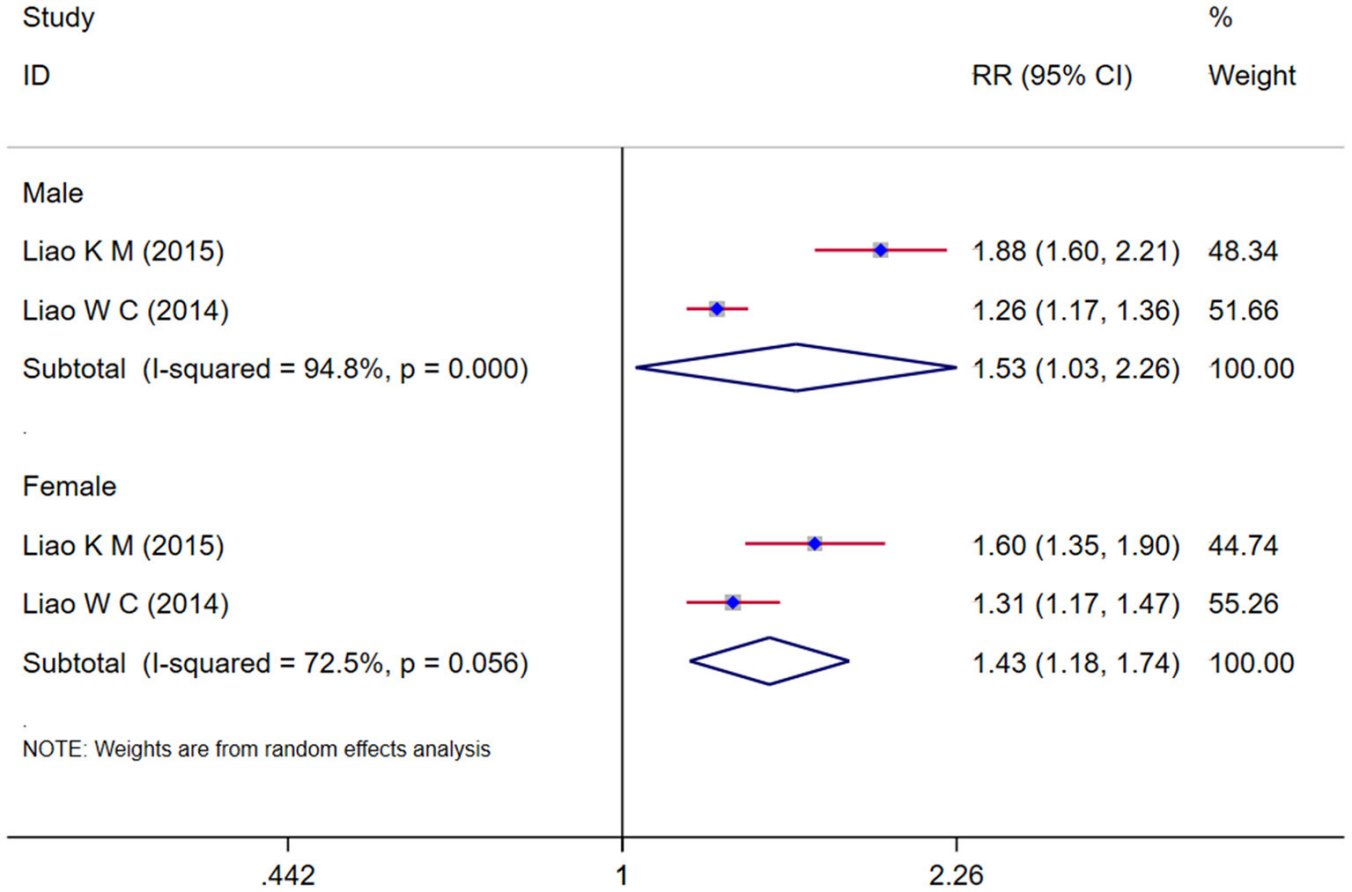
Subgroup analysis by gender evaluating the risk of dementia in COPD patients.

Two studies (Liao et al., 2015a,b) assessed the relationship between the gender of COPD and the risk of dementia. The subgroup analysis result showed that there is no difference between male and female, both sex patients with COPD had an increased risk of dementia (male: *RR* = 1.53, 95% *CI* = 1.03 ~ 2.26, *I*^2^ = 94.8%, *z* = 2.12, *p* = 0.034*;* female: *RR* = 1.43, 95% *CI* = 1.18 ~ 1.74, *I*^2^ = 72.5%, *z* = 3.62, *p* < 0.001) (Figure 3).

### Age of COPD and risk of dementia

**Figure 4:**
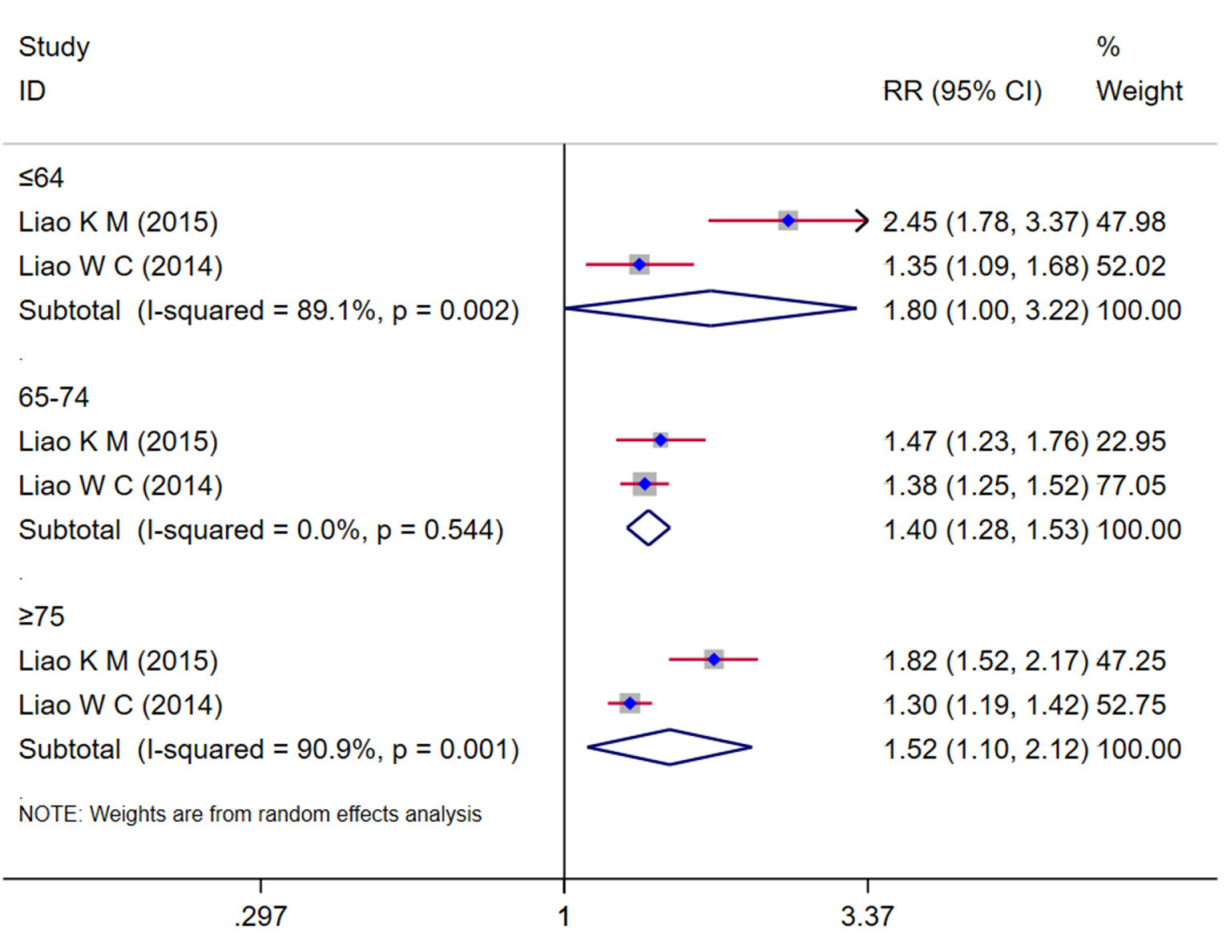
Subgroup analysis by age group evaluating the risk of dementia in COPD patients.

Two studies (Liao et al., 2015a,b) assessed the relationship between the age of COPD and the risk of dementia. The subgroup analysis result (Figure 4) showed that the COPD patients younger than 65 years were significant associated with a risk of dementia (*RR* = 1.80, 95% *CI* = 1.00~3.22, *I*^2^ = 89.1%, *z* = 1.97, *p* = 0.049), and the COPD patients aged between 65 and 74 found an increase in the risk of dementia (*RR* = 1.40, 95% *CI* = 1.28 ~ 1.53, *I*^2^ = 0.00%, *z* = 7.69, *p* < 0.001), the COPD patients with 75 years or older also showed a significant increase in the risk of dementia (*RR* = 1.52, 95% *CI* = 1.10 ~ 2.12, *I*^2^ = 90.0%, *z* = 2.51, *p* = 0.012).

### Association between COPD and cognitive impairment

**Figure 5:**
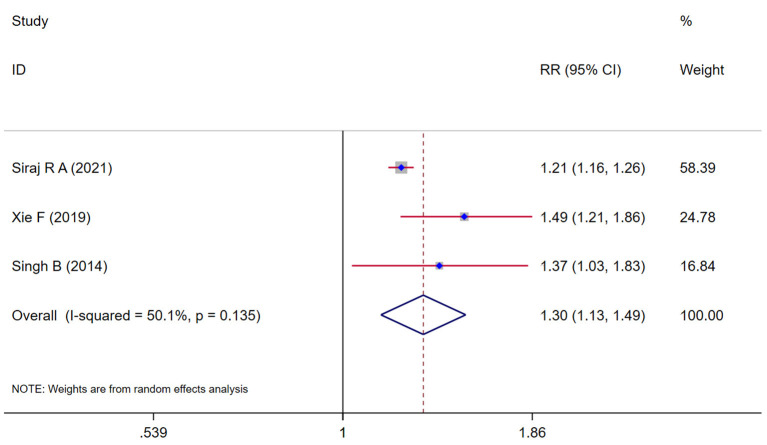
Forest plot showing the effect of COPD on cognitive impairment.

Three studies (Singh et al., 2014; Xie and Xie, 2019; Siraj et al., 2020) evaluated the risk of cognitive impairment in COPD and the pooling analysis shows that COPD was associated with an increased risk of cognitive impairment (*RR* = 1.30, 95% *CI* = 1.13 ~ 1.49, *I*^2^ = 50.1%, *z* = 3.72, *p* < 0.001; Figure 5).

## Discussion

Six cohort studies (Singh et al., 2014; Liao et al., 2015a,b; Xie and Xie, 2019; Siraj et al., 2020; Tao et al., 2020) were included in this meta-analysis, covering 428,030 individuals. We compiled their evidence on the association between COPD and the risks of dementia or cognitive and found a significant increase in the risk of dementia or cognitive impairment among patients with COPD.

In comparison with the previous study, a meta-analysis study (Wang et al., 2019) showed that COPD patients faced a higher risk of dementia, consistent with the results of our study, which suggest that patients with COPD have a higher risk of developing dementia compared to those without COPD. However, they obtained an *HR* for the risk of dementia which was higher than that observed in our study. The reason may be that we included more updated evidence from different countries and regions, in which a new cohort study from the UK (Siraj et al., 2020) provided different conclusions, and these research data were incorporated into statistical analysis and had an impact on the results. Further subgroup analysis revealed a high risk of dementia in both male and female patients with COPD; and in terms of age, COPD patients of different ages are at significant risk of dementia. But unlike older COPD patients, these younger than 64 years revealed a more significant association with dementia, which suggested a greater risk of developing dementia in younger patients with COPD. Notably, although older age was associated with a higher risk of dementia (Li et al., 2018), younger patients with COPD significantly affects this course. Research has revealed the correlation between PM2·5 exposure and COPD prevalence in young people is stronger than that in middle-aged and elderly people, and the detrimental effects on lung development may be greater (Wang et al., 2018). It can be assumed that exposure to COPD-related risk factors affects the age of onset, and has a greater impact on young people, leading to a higher risk of dementia. Since only two studies were included in the subgroup analysis, which may reduce statistical efficiency, and these studies were from the same region, the results are less representative. Therefore, more large-scale epidemiological evidence is needed to understand the age-related risks of dementia in patients with COPD.

Consistent with previous findings (Zhang et al., 2016), it is noteworthy that a significant association was found between COPD and the risk of cognitive impairment in our meta-analysis. They included 14 studies, including three cohort studies as well as 11 cross-sectional studies. According to their results, COPD patients had a higher risk of cognitive dysfunction than controls, which was slightly higher than that observed in our study. The reason may be that we included different types and numbers of studies and used different statistical methods. Furthermore, a prior population-based study (Rusanen et al., 2013), including 2,000 participants, with more than 25 years of follow-up, also indicated that midlife COPD was associated with an almost two-fold risk of mild cognitive impairment (MCI) and dementia later in life.

Therefore, based on the evidence from this study and other relevant evidence, we considered that patients with COPD have a higher risk of developing dementia or cognitive impairment compared to those without COPD. Although the exact etiological link between COPD and cognitive impairment remains unknown, a review (Kakkera et al., 2018) indicated that there were several mechanisms proposed for the association of COPD with higher rates of cognitive impairment, including oxidative stress, tissue hypoxemia, inactive state, and systemic inflammatory state. Oxidative stress caused by systemic inflammation and hypoxia was considered to be one of the causes of dementia, which may be a mechanism that links cardiopulmonary pathology with neurocognitive impairment (Cheung et al., 2018). Inflammatory markers such as the levels of interleukin (IL)-6, C-reactive protein (CRP), and tumor necrosis factor-alpha (TNF-α) also have been reported to be elevated in patients with COPD (Deveci et al., 2010; Custodero et al., 2022). In addition, a study (Ranzini et al., 2020) showed patients with COPD may show cerebral perfusion alterations as a consequence of hypoxemia, which is an abnormal decrease in oxygen in the blood, and these changes could lead to cognitive impairment. Neuroimaging provides further evidence of a relationship between lung function and cognition. A study (Lv et al., 2020) revealed that abnormal static and dynamic local-neural activities in the basal ganglia and parahippocampal/hippocampal cortex in COPD patient was related to poor lung function and semantic-memory impairments. COPD patients with cognitive impairment showed greater disability, risk of exacerbation, and poorer medication compliance, and a diagnosis of MCI was considered a precursor to a diagnosis of dementia. Although progression to dementia in MCI patients occurs over many years (Reisberg et al., 2010), COPD may exacerbate this process, which may provide some reference for clinical early preventive treatment.

Six studies (Singh et al., 2014; Liao et al., 2015a,b; Xie and Xie, 2019; Siraj et al., 2020; Tao et al., 2020) were included in this study, only one (Siraj et al., 2020) showed that COPD had no increased risk of dementia. We speculated that this may be related to the following reasons: the sample size included between the COPD and control varies greatly, which may influence the results of the statistical analysis. In addition, the different confounders adjusted by each study could be another reason. Furthermore, the included cases were from the UK, a highly developed country where the economic culture and the level of medical care may be an important factor in the risk of dementia. It is worth considering the heterogeneity of the included studies was large in this meta-analysis, which may be attributable to differences in sample size between included studies, inconsistent diagnostic criteria for COPD and dementia or cognitive impairment, and different confounders adjusted, etc.

There were some limitations in this meta-analysis. Firstly, the number of eligible studies is small, which prevented further subgroup analyses on the characteristics that influence the outcome, such as comorbidities, types of dementia or cognitive impairment, and different regions. In addition, the included studies did not provide data related to chronic obstructive pulmonary classification, therefore we were unable to perform relevant subgroup analyses to explain the risk of dementia or cognitive impairment in patients with different degrees of chronic obstructive pulmonary disease. Furthermore, as four of the six studies reflect data collected in patients living in China, the overall findings may not be applicable to patients with COPD living in other countries. Finally, the included studies showed significant heterogeneity in terms of population size, follow-up years, diagnosis codes, and confounders adjusted. Therefore, the potential impact of heterogeneity on the results should be considered when interpreting our results.

## Conclusion

Our findings showed a significant increase in the risk of dementia or cognitive impairment among patients with COPD and this risk is not affected by gender, but it seems to be affected by age. COPD patients of different ages indicated different degrees of dementia risk, and this risk was more pronounced in patients younger than 65 years. However, due to the small number of studies included in subgroup analysis and high heterogeneity, which need to be treated with caution when interpreting our results.

## Data availability statement

The datasets presented in this study can be found in online repositories. The names of the repository/repositories and accession number(s) can be found in the article/Supplementary material.

## Author contributions

JL, XL, and JW conceived the study. JW, XL, SL, DZ, SZ, HZ and JL performed literature search, selection, and data extraction. XL, JW, and SZ contributed to data interpretation and analysis. XL, SL, DZ, HZ, and JL revised the manuscript. All authors reviewed and approved the manuscript.

## Funding

This work was funded by the Chinese Medicine Inheritance and Innovation Hundred and Ten Million Talent Project—Chief Scientist of Qi-Huang Project [(2020) No. 219]; Zhong-Yuan Scholars and Scientists Project (No. 2018204); and Characteristic Backbone Discipline Construction Project of Henan Province (STG-ZYX03-202123).

## Conflict of interest

The authors declare that the research was conducted in the absence of any commercial or financial relationships that could be construed as a potential conflict of interest.

## Publisher's note

All claims expressed in this article are solely those of the authors and do not necessarily represent those of their affiliated organizations, or those of the publisher, the editors and the reviewers. Any product that may be evaluated in this article, or claim that may be made by its manufacturer, is not guaranteed or endorsed by the publisher.
