# Supplementary material for: Risk of dementia or cognitive impairment in COPD patients: A meta-analysis of cohort studies
Source: Front Aging Neurosci. 2022 Sep 9;14:962562. doi: 10.3389/fnagi.2022.962562 (PMC9500359; doi:10.3389/fnagi.2022.962562)
Supplement: Supplementary file 1 [file Data_Sheet_1.PDF]

Supplementary table 1

Details of the Literature Search Strategy

(1) PubMed

Search	Query	Items found
#1	Pulmonary Disease, Chronic Obstructive[MeSH]	62,810
#2	(((((((((Pulmonary Disease, Chronic Obstructive[Title/Abstract]) OR (Chronic obstructive pulmonary disease[Title/Abstract])) OR (COPD[Title/Abstract])) OR (COAD[Title/Abstract])) OR (chronic obstructive lung disease[Title/Abstract])) OR (chronic obstructive airway disease[Title/Abstract])) OR (chronic obstructive respiratory disease[Title/Abstract])) OR (Chronic bronchitis[Title/Abstract])) OR (chronic emphysema[Title/Abstract])) OR (Chronic Airflow Obstruction[Title/Abstract]))	85,522
#3	#1OR#2	105,018
#4	(((cognition disorders[MeSH]) OR (Dementia[Mesh])) OR (Alzheimer Disease[Mesh]))	262,200
#5	(((((((((((cognitive defect[Title/Abstract]) OR (cognitive decline[Title/Abstract])) OR (cognitive deficit[Title/Abstract])) OR (cognitive dysfunction[Title/Abstract])) OR (cognitive impairment[Title/Abstract])) OR (mild cognitive impairment[Title/Abstract])) OR (neurocognitive disorder[Title/Abstract])) OR (memory impairment[Title/Abstract])) OR (vascular dementia[Title/Abstract])) OR (multiinfarct dementia[Title/Abstract]))	254,538

	OR (alzheimer[Title/Abstract])) OR (alzheimer’s disease[Title/Abstract]))	
#6	#4 OR #5	369,176
#7	#3 AND #6	849

(2) Embase

Search	Query	Items found
#1	'chronic obstructive lung disease'/exp	158,102
#2	'Pulmonary Disease, Chronic Obstructive':ti,kw,ab OR 'Pulmonary Disease, Chronic Obstructive':ti,kw,ab OR 'Chronic obstructive pulmonary disease':ti,kw,ab OR COPD:ti,kw,ab OR COAD:ti,kw,ab OR 'chronic obstructive airway disease':ti,kw,ab OR 'chronic obstructive respiratory disease':ti,kw,ab OR 'Chronic bronchitis':ti,kw,ab OR 'chronic emphysema':ti,kw,ab OR 'Chronic Airflow Obstruction':ti,kw,ab	141,887
#3	#1 OR #2	195,133
#4	'cognitive defect'/exp	558.602
#5	'dementia'/exp	405.834
#6	'alzheimer disease'/exp	224,141

#7	'Cognition Disorders':ti,kw,ab OR 'cognitive decline':ti,kw,ab OR 'cognitive deficit':ti,kw,ab OR 'cognitive dysfunction':ti,kw,ab OR 'cognitive impairment':ti,kw,ab OR 'mild cognitive impairment':ti,kw,ab OR 'neurocognitive disorder':ti,kw,ab OR 'memory impairment':ti,kw,ab OR 'vascular dementia':ti,kw,ab OR 'multiinfarct dementia':ti,kw,ab OR alzheimer:ti,kw,ab OR 'Alzheimers disease':ti,kw,ab	362,921
#8	#4 OR #5 OR #6 OR #7	627.423
#9	#3 AND #8	4.789

(3) Cochrane Library

**Search**	**Query**	**Items found**
#1	MeSH descriptor: [Pulmonary Disease, Chronic Obstructive] explode all trees	6247
#2	(Pulmonary Disease, Chronic Obstructive):ti,ab,kw OR (Chronic obstructive pulmonary disease):ti,ab,kw OR (COPD):ti,ab,kw OR (COAD):ti,ab,kw OR (chronic obstructive lung disease):ti,ab,kw	22753
#3	(chronic obstructive airway disease):ti,ab,kw OR (chronic obstructive respiratory disease):ti,ab,kw OR (Chronic bronchitis):ti,ab,kw OR (chronic emphysema):ti,ab,kw OR (Chronic Airflow Obstruction):ti,ab,kw	10794
#4	#1 OR #2 OR #3	25070
#5	MeSH descriptor: [Cognition Disorders] explode all trees	5934

#6	MeSH descriptor: [Dementia] explode all trees	6567
#7	MeSH descriptor: [Alzheimer Disease] explode all trees	3706
#8	(cognitive defect):ti,ab,kw OR (cognitive decline):ti,ab,kw OR (cognitive deficit):ti,ab,kw OR (cognitive dysfunction):ti,ab,kw OR (cognitive impairment):ti,ab,kw	31717
#9	(mild cognitive impairment):ti,ab,kw OR (neurocognitive disorder):ti,ab,kw OR (memory impairment):ti,ab,kw OR (vascular dementia):ti,ab,kw OR (multiinfarct dementia):ti,ab,kw	13775
#10	(alzheimer):ti,ab,kw OR (Alzheimer's disease):ti,ab,kw	12249
#11	#5 OR #6 OR #7 OR #8 OR #9 OR #10	45613
#12	#4 AND #11	247

(4) Web of Science core collection

**Search**	**Query**	**Items found**
#1	TS=(Pulmonary Disease, Chronic Obstructive) OR TS=(Pulmonary Disease, Chronic Obstructive) OR TS=(Chronic obstructive pulmonary disease) OR TS=(COPD) OR TS=(COAD) OR TS=(chronic obstructive lung disease) OR TS=(chronic obstructive airway disease) OR TS=(chronic obstructive respiratory disease) OR TS=(Chronic bronchitis) OR TS=(chronic emphysema) OR TS=(Chronic Airflow Obstruction)	162,527

#2	TS=(cognition disorders) OR TS=(Dementia) OR TS=(Alzheimer Disease) OR TS=(cognitive defect) OR TS=(cognitive decline) OR TS=(cognitive deficit) OR TS=(cognitive dysfunction) OR TS=(cognitive impairment) OR TS=(mild cognitive impairment) OR TS=(neurocognitive disorder) OR TS=(memory impairment) OR TS=(vascular dementia) OR TS=(multiinfarct dementia) OR TS=(alzheimer) OR TS=(alzheimer’s disease)	729,489
#3	#1 AND #2	2,395
