# Supplementary material for: Risk of dementia or cognitive impairment in COPD patients: A meta-analysis of cohort studies
Source: Front Aging Neurosci. 2022 Sep 9;14:962562. doi: 10.3389/fnagi.2022.962562 (PMC9500359; doi:10.3389/fnagi.2022.962562)
Supplement: Supplementary file 2 [file Data_Sheet_2.PDF]

Supplementary table 2

Characteristics of Excluded Studies

Author, Year	Tittle	Reasons
Liao K, et al 2019	The Association Between Dementia and COPD: A Nationwide Population Based, Propensity Score Matched Cohort Study.	Conference Abstract
Siraj R, et al 2018	Dementia in patients with chronic obstructive pulmonary disease (COPD) A UK based population-based study.	
Carpenter C 2017	The prevalence of cognitive impairment in COPD	
Samareh Fekri M, et al 2017	Cognitive Impairment among Patients with Chronic Obstructive Pulmonary Disease Compared to Normal Individuals.	case-control study
Metwally M, et al 2017	Evaluation of cognitive function in COPD: A role of p-300 event-related potentials.	
Zhou G, et al 2012	Association of chronic obstructive pulmonary disease with cognitive decline in very elderly.	
Feng AF, et al 2016	Association between chronic obstructive pulmonary disease and the risk for mild cognitive impairment: a retrospective cohort study.	Failed to download full text

Yohannes AM, et al 2021	Association of mild cognitive impairment and characteristic of COPD and overall health status in a cohort study.	Without interest outcome
Hoth K, et al 2020	Risk for cognitive impairment in smokers with preserved ratio impaired spirometry-an analysis of the COPD gene cohort.	
Krauskopf K, et al 2013	COPD and cognitive impairment: Associations with inhaler technique.	
Rusanen M, et al 2013	Chronic obstructive pulmonary disease and asthma and the risk of mild cognitive impairment and dementia: a population based CAIDE study.	
Thakur N, et al 2010	COPD and cognitive impairment: the role of hypoxemia and oxygen therapy.	ongoing study
