# Supplementary figures and images for: Risk of dementia or cognitive impairment in COPD patients: A meta-analysis of cohort studies
Source: Front Aging Neurosci. 2022 Sep 9;14:962562. doi: 10.3389/fnagi.2022.962562 (PMC9500359; doi:10.3389/fnagi.2022.962562)

Supplementary table 3

Sensitivity analyses showing the effect of COPD on dementia

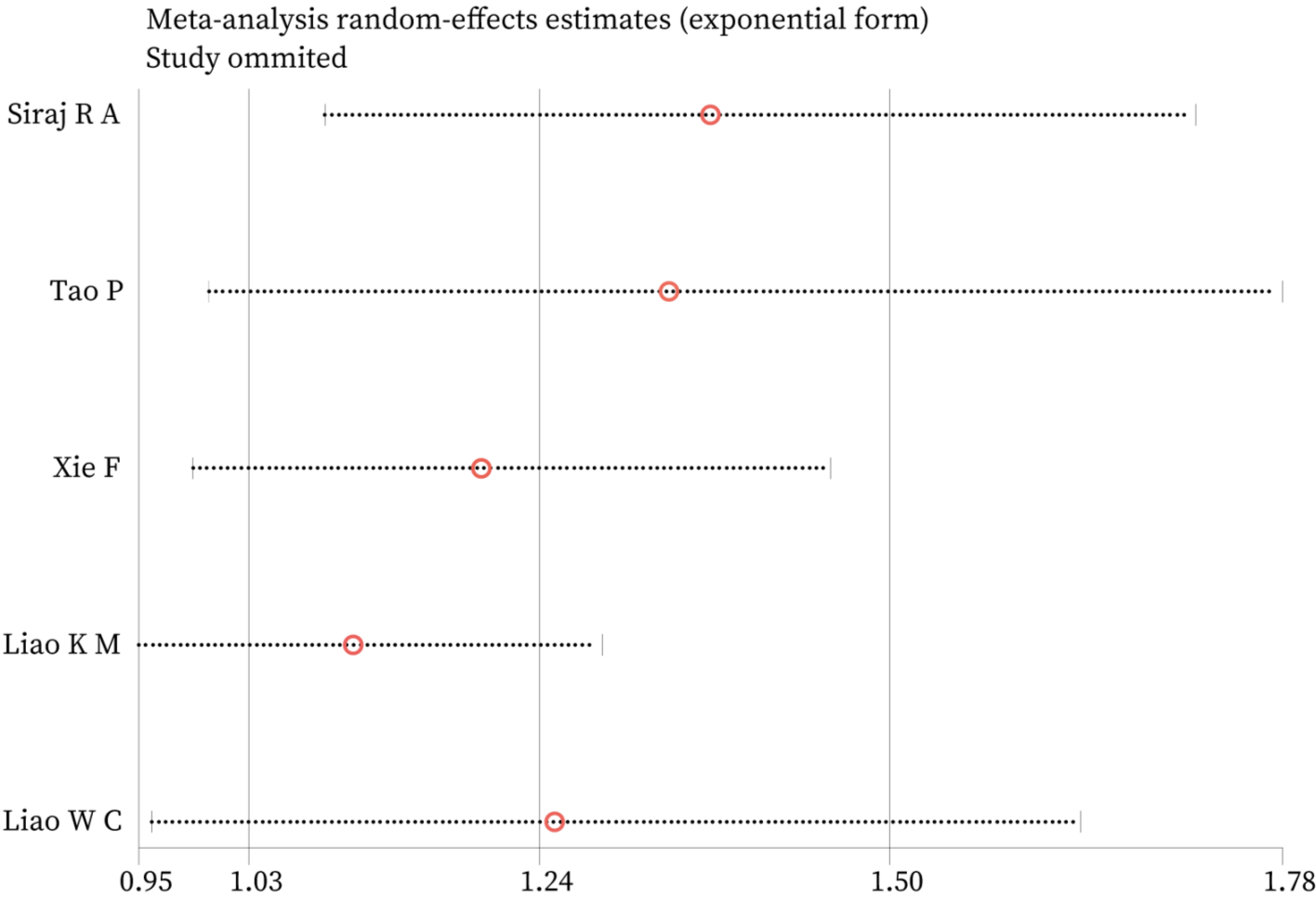

Supplement: Supplementary file 3 [file Data_Sheet_3.PDF]
